# Simultaneous Clear Cell Sarcomas of the Duodenum and Jejunum

**DOI:** 10.1155/2016/1534029

**Published:** 2016-06-07

**Authors:** Maitham A. Moslim, Gavin A. Falk, Michael Cruise, Gareth Morris-Stiff

**Affiliations:** ^1^Department of HPB Surgery, Digestive Disease Institute, Cleveland Clinic Foundation, 9500 Euclid Avenue, Cleveland, OH 44195, USA; ^2^Department of Anatomic Pathology, Cleveland Clinic Foundation, 9500 Euclid Avenue, Cleveland, OH 44195, USA

## Abstract

Clear cell sarcoma (CCS) is an uncommon tumor that usually presents as an extremity mass but can rarely manifest as a gastrointestinal tumor with a diverse spectrum of symptoms, most commonly related to a mass effect or ulceration. Herein we report a case in which two separate tumors, one in the duodenum and the other in the jejunum, present concurrently. The subject presented with symptomatic anemia and underwent imaging and endoscopic studies that culminated in the discovery of the two lesions. He subsequently underwent operative treatment with resection of both tumors and made an unremarkable recovery. The resection specimen consisted of two separate clear cell sarcomas with negative margins. Under microscopic evaluation, they demonstrated nested growths of epithelioid cells with scattered spindled cells infiltrating the enteric wall. The neoplastic cells were positive for S100 with scattered expression of Melan A. Florescence in situ hybridization revealed a translocation at the EWRS1 locus. He was disease-free for 30 months following the procedure; then he developed a rapidly progressing metastatic disease with subsequent death 4 months later.

## 1. Background

Clear cell sarcoma (CCS) is a rare distinctive soft tissue sarcoma, known to originate from neural crest cells, that has been classified as a variant of melanoma since it was first reported in 1965 [1]. Its usual presentation is as a lower extremity tumor; however it has been rarely reported to manifest within the gastrointestinal (GI) tract. The diagnosis of CCS is often missed as a result of its low incidence and frequent misdiagnosis as malignant melanoma as a result of the fact that pathologically it exhibits melanocytic differentiation [2]. The early diagnosis of CCS is key since surgical resection can lead to long-term survival.

## 2. Case Presentation

**Figure 1:**
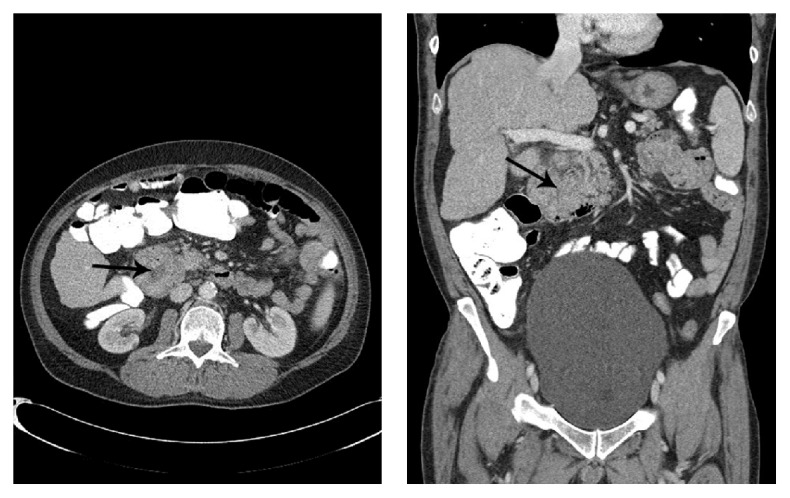
Axial and coronal CT of the abdomen demonstrating a 4.7 cm heterogeneously enhancing mass (arrow) involving the second portion of the duodenum. The lumen of the duodenum is compressed by the mass.

A 57-year-old gentleman presented to his local hospital with several months' history of fatigue, dizziness, and fall episodes two weeks prior to his presentation to our institution. He also reported a 15-pound weight loss over the prior 3 weeks but denied abdominal pain or any symptoms suggestive of gastrointestinal bleeding. His hemoglobin was 7.4 g/dL with a microcytic picture: mean corpuscular volume (MCV) of 76.9 fL (normal range is 80–100 fL), and a mean corpuscular hemoglobin (MCH) of 25.6 pg (28–34 pg). He underwent esophagogastroduodenoscopy (EGD) that revealed an antral gastric ulcer and a 3 cm nodular, ulcerated, polypoid mass in the second portion of the duodenum, occupying 50% of the lumen. The endoscopist was unable to advance the scope past the duodenal mass. Multiple cold forceps biopsies were obtained and demonstrated atypical epithelioid cells with large nuclei, prominent nucleoli, and readily identifiable mitotic activity without a glandular component. A computerized tomography (CT) scan of the abdomen demonstrated a 4.7 cm heterogeneously enhancing mass within the second portion of the duodenum (Figure 1). His past medical history consisted only of rheumatoid arthritis and Graves' disease that had been treated with radioactive iodine therapy 6 years previously, and he had a smoking history of 40 pack years.

He was transferred to our institution for ongoing management. He underwent a contrast-enhanced triple phase CT scan of the chest, abdomen, and pelvis, which revealed no evidence of metastatic disease but did show evidence of liver nodularity consistent with cirrhosis, together with splenomegaly and splenic venous varices. There was a significant circumferential wall thickening involving the duodenum without any vascular encroachment or invasion. Hepatitis serologies were sent and returned positive for hepatitis C antibodies whilst hepatitis C ribonucleic acid polymerase chain reaction test (RNA PCR) was negative. His Child-Pugh (CP) score was calculated as 7 and he was classed as CP B.

The patient was taken to the operative room and underwent an exploratory laparotomy at which no evidence of metastatic disease was evident. Nodularity of the liver and sequelae of portal hypertension were observed consistent with cirrhosis. A large duodenal tumor was identified together with a separate proximal jejunal tumor. A pancreatoduodenectomy was performed with a separate resection of the jejunal mass as the tumor was several arcades distal to the duodenal mass. The reconstruction of the duodenal resection was fashioned by means of pancreaticojejunostomy, end-to-side choledochojejunostomy, and gastrojejunostomy, whilst an end-to-end reconstruction was performed following the jejunal resection. A liver biopsy was also performed.

Following the surgery, he recovered well in the surgical intensive care unit and was transferred to the regular nursing floor the following day. He was commenced on total parenteral nutrition due to severe chronic malnutrition with a gradual transition to oral diet. The drain fluid analysis showed no pancreatic leak.

**Figure 2:**
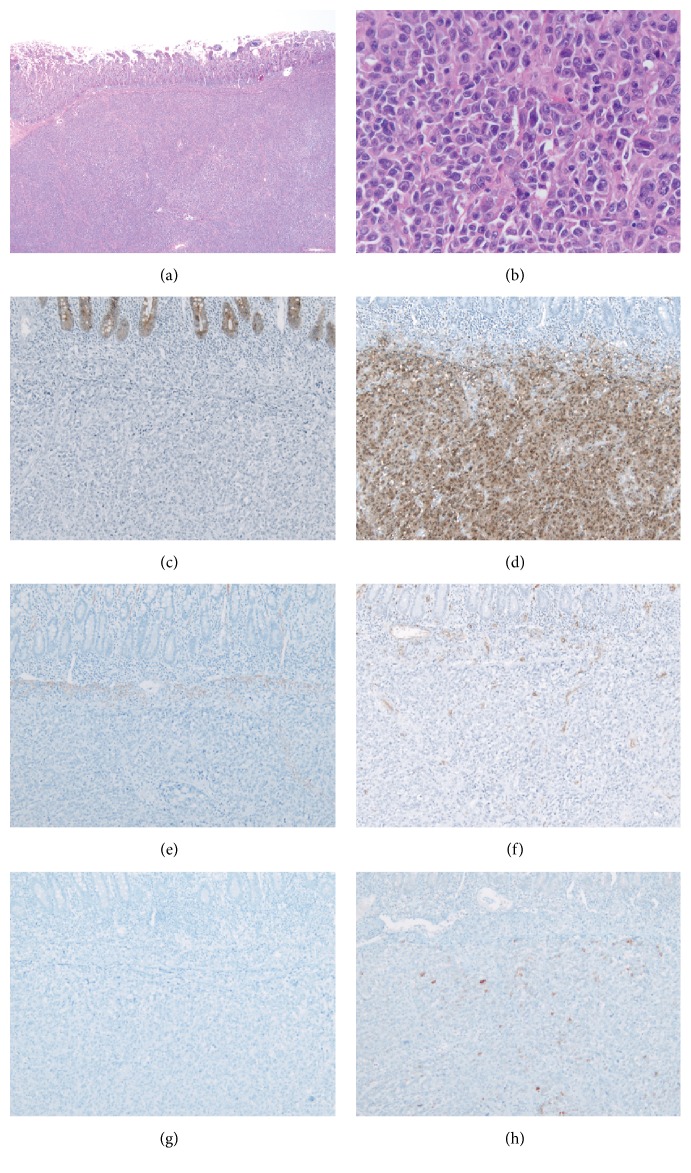
The tumor demonstrates a nested growth pattern underlying the small bowel mucosa: (a) HE 20x, original magnification. The neoplastic cells are large eosinophilic cells with prominent cherry red nucleoli: (b) H&E 400x, original magnification. The neoplastic cells are positive for S100 (d) and Melan A (h) but negative for pan-cytokeratin AE1/3 (c), desmin (e), CD34 (f), and HMB45 (g).

The resection specimen consisted of two clear cell sarcomas both exhibiting negative margins. The duodenal tumor measured 5.5 × 2.5 × 2.5 cm and the jejunal tumor 7.5 × 5.5 × 2.0 cm. The mass was white-tan and rubbery with a central area of necrosis. At low power the tumor was characterized by a nested growth pattern involving the subserosal adipose tissue, submucosa, and demonstrated infiltration into the lamina propria. The neoplastic cells were epithelioid with only scattered spindled cells. The cells were large with eosinophilic cytoplasm, pleomorphic nuclei, and distinct cherry red macronucleoli. Mitotic figures were readily identified. There was no epithelial dysplasia or mucosal precursor lesions identified. A panel of immunostains demonstrated that the neoplastic cells were strongly positive for S100 with scattered strong expression of Melan A. The neoplastic cells were negative for pan-cytokeratin (AE1/3 and CAM5.2), CK7, CK20, CDX-2, chromogranin, synaptophysin, TTF-1, desmin, smooth muscle actin, CD34, and HMB-45. To exclude a metastatic melanoma, florescence in situ hybridization for EWSR1 (22q12) gene rearrangement was performed and the tumour was found to have a translocated EWRS1 locus. The morphologic, immunohistochemical, and FISH results supported the diagnosis of clear cell sarcoma (Figure 2). The liver core biopsy confirmed cirrhosis.

Two of 31 lymph nodes from the duodenal resection specimen were positive for tumor involvement whilst 0 of 5 lymph nodes from the jejunal specimen exhibited tumor infiltration. His tumor was staged as T2b, N1, and M0 (Stage III) for the duodenal CCS and T2b, N0, and M0 (Stage III) for the jejunal tumor. The mitotic rate was 54 per 10 high-power fields (HPFs) and both tumors were assigned grade 3.

Following confirmation of the diagnosis, he underwent a full body examination, with no evidence of pigmented lesions seen.

He presented to our clinic for his postoperative visit and had made an excellent recovery. He also followed up with the oncology team but no adjuvant chemotherapy was commenced due to a lack of evidence based benefit and cirrhosis. He was therefore assigned to regular cross-sectional imaging studies and was disease-free for 30 months following the procedure; then he developed a rapidly progressing metastatic disease with subsequent death 4 months later.

## 3. Discussion

CCS was originally described in 1965 and was known as “malignant melanoma of soft parts” in view of its melanocytic differentiation [1]. It typically involves tendons and aponeuroses of the extremities, most commonly the foot and ankle. CCS manifests as a slowly growing, often painful mass [1]. Primary GI CCSs are extremely uncommon with a predilection for the jejunum [*n* = 11] and ileum [*n* = 8] (70% of cases), whilst the stomach, colon, pancreas, and duodenum are rarely involved [2–5]. Soft tissue CCS is usually more common in young females; however gastrointestinal CCS predominates in young to middle-aged adults with equal gender distribution [2]. Patients usually present with abdominal pain, bowel obstruction, GI bleeding, nausea, or diarrhea.

The limited literature on gastrointestinal CCS does not contain any cases of multiple synchronous tumors and to date there is only a single case of duodenal CCS reported [6]. Ekfors et al. reported the case of a 38-year-old male who presented with epigastric discomfort and nausea. Further workup revealed an ulcer in the descending duodenum with a biopsy showing suspicion for carcinoma. However a microscopic examination of the resected specimen confirmed a pathologic pattern similar to our case, although the mitotic activity was low.

CCS is considered an aggressive malignant neoplasm with unfavorable prognosis, and most patients deceased within 2 years of diagnosis. The overall poor prognosis is related to the vague clinical presentation and thereby a delayed diagnosis [3, 4, 7, 8]. Gastrointestinal CCS has an aggressive clinical course with frequent regional and distant metastases at presentation. Common destinations of metastases include lymph nodes and liver. At the time of presentation most patients demonstrate regional lymphadenopathy (64.7%) and hepatic metastases (60%), and mesenteric metastases are also frequently observed [2]. The natural history of CCS also includes transmural infiltration of the bowel wall with a tendency to ulcerate and invade into the mesentery or adjacent organs and/or disseminate throughout the peritoneal cavity. Ultimately, all cases require a multidisciplinary approach involving radiology, interventional endoscopy, gastrointestinal surgery, and oncology; however surgical resection is often not a curative treatment due to the delayed diagnosis and the presence of metastatic disease. There is no documented role for adjuvant chemotherapy in nonmetastatic disease providing the resistance of this neoplasm to the standard chemotherapeutic regimens [8].

CCS of the GI tract has a very similar histologic appearance to metastatic melanoma, and it also needs to be differentiated from GI stromal tumours (GIST) and poorly differentiated papillary adenocarcinoma [9]. Histopathological examination of CCS reveals the archetypal features of eosinophilic spindle cells arranged in bundles or nests intercalated by dense fibrous septa [10]. On the cellular level, classical features include pleomorphic cellularity, high mitotic index, and presence of melanosomes, with this combination of features being reported in half the cases of CCS reported in the literature. Immunohistochemical staining of CCS reveals positivity for the S100 protein as well as melanocyte-specific markers, with this combination of staining allowing CCS to be distinguished from malignant melanoma histologically. Markers specific for neuroendocrine differentiation such as chromogranin-A are negative, as are markers indicative of GIST including CD34 and desmin [11, 12].

In addition, cytogenetic studies provide important data to distinguish CCS from melanoma. In CCS, a chromosomal translocation at t(12;22)(q13;q12), encoding the unique EWSR1/ATF1 fusion transcript, is present in 75% of cases, and less commonly a t(2;22)(q34;q12) translocation may be seen encoding the unique EWSR1/CREB1 fusion transcript. To date, these translocations have never been observed in malignant melanoma [12–17]. Reverse-transcription polymerase chain reaction (RT-PCR) and fluorescence in situ hybridization (FISH) are now considered the gold standard laboratory tests to examine the specimen for t(12;22) translocation. CCS also lack BRAF mutations which are commonly found in melanoma [3].

## Additional Points

This paper is summarized as follows:

- Clear cell sarcoma is a rare variant of melanoma of the gastrointestinal tract.
- Fluorescence in situ hybridization (FISH) is the gold standard test to detect the t(12;22) translocation that is pathognomonic of clear cell sarcoma.
- Most patients deceased within 2 years of diagnosis.
- The early diagnosis is essential as surgical resection can lead to long-term survival.

## References

[1] Enzinger F. M. (1965). Clear-cell sarcoma of tendons and aponeuroses. An analysis of 21 cases. *Cancer*.

[2] Mehta A., Brownson K., Massarani R., Esposito T., Abood G., Shoup M. (2013). Clear cell sarcoma of the jejunum—surgical management in two patients with review of the literature. *Case Reports in Clinical Medicine*.

[3] Antonescu C. R., Nafa K., Segal N. H., Dal Cin P., Ladanyi M. (2006). EWS-CREB1. A recurrent variant fusion in clear cell sarcoma—association with gastrointestinal location and absence of melanocytic differentiation. *Clinical Cancer Research*.

[4] Stockman D. L., Miettinen M., Suster S. (2012). Malignant gastrointestinal neuroectodermal tumor: clinicopathologic, immunohistochemical, ultrastructural, and molecular analysis of 16 cases with a reappraisal of clear cell sarcoma-like tumors of the gastrointestinal tract. *American Journal of Surgical Pathology*.

[5] Abdulkader I., Cameselle-Teijeiro J., de Alava E., Ruiz-Ponte C., Used-Aznar M. M., Forteza J. (2008). Intestinal clear cell sarcoma with melanocytic differentiation and extraskeletal myxoid chondrosarcoma rearrangement: report of a case. *International Journal of Surgical Pathology*.

[6] Ekfors T. O., Kujari H., Isomäki M. (1993). Clear cell sarcoma of tendons and aponeuroses (malignant melanoma of soft parts) in the duodenum: the first visceral case. *Histopathology*.

[7] Zambrano E., Reyes-Mugica M., Franchi A., Rosai J. (2003). An osteoclast-rich tumor of the gastrointestinal tract with features resembling clear cell sarcoma of soft parts: reports of 6 cases of a GIST simulator. *International Journal of Surgical Pathology*.

[8] Kosemehmetoglu K., Folpe A. L. (2010). Clear cell sarcoma of tendons and aponeuroses, and osteoclast-rich tumour of the gastrointestinal tract with features resembling clear cell sarcoma of soft parts: a review and update. *Journal of Clinical Pathology*.

[9] Lyle P. L., Amato C. M., Fitzpatrick J. E., Robinson W. A. (2008). Gastrointestinal melanoma or clear cell sarcoma? Molecular evaluation of 7 cases previously diagnosed as malignant melanoma. *American Journal of Surgical Pathology*.

[10] Taminelli L., Zaman K., Gengler C. (2005). Primary clear cell sarcoma of the ileum: an uncommon and misleading site. *Virchows Archiv*.

[11] Rosai J. (2005). Clear cell sarcoma and osteoclast-rich clear cell sarcoma-like tumor of the gastrointestinal tract: one tumor type or two? Melanoma or sarcoma?. *International Journal of Surgical Pathology*.

[12] Wang W.-L., Mayordomo E., Zhang W. (2009). Detection and characterization of EWSR1/ATF1 and EWSR1/CREB1 chimeric transcripts in clear cell sarcoma (melanoma of soft parts). *Modern Pathology*.

[13] Covinsky M., Gong S., Rajaram V., Perry A., Pfeifer J. (2005). *EWS-ATF1* fusion transcripts in gastrointestinal tumors previously diagnosed as malignant melanoma. *Human Pathology*.

[14] Lagmay J. P., Ranalli M., Arcila M., Baker P. (2009). Clear cell sarcoma of the stomach. *Pediatric Blood and Cancer*.

[15] Panagopoulos I., Mertens F., Isaksson M., Mandahl N. (2005). Absence of mutations of the BRAF gene in malignant melanoma of soft parts (clear cell sarcoma of tendons and aponeuroses). *Cancer Genetics and Cytogenetics*.

[16] Panagopoulos I., Mertens F., Dêbiec-Rychter M. (2002). Molecular genetic characterization of the EWS/ATF1 fusion gene in clear cell sarcoma of tendons and aponeuroses. *International Journal of Cancer*.

[17] Langezaal S. M., Graadt Van Roggen J. F., Cleton-Jansen A. M., Baelde J. J., Hogendoorn P. C. W. (2001). Malignant melanoma is genetically distinct from clear cell sarcoma of tendons and aponeurosis (malignant melanoma of soft parts). *British Journal of Cancer*.

